# Extraction of Rice Heavy Metal Stress Signal Features Based on Long Time Series Leaf Area Index Data Using Ensemble Empirical Mode Decomposition

**DOI:** 10.3390/ijerph14091018

**Published:** 2017-09-06

**Authors:** Lingwen Tian, Xiangnan Liu, Biyao Zhang, Ming Liu, Ling Wu

**Affiliations:** School of Information Engineering, China University of Geoscience, Beijing 100083, China; tianlw@cugb.edu.cn (L.T.); Zhangbycn@cugb.edu.cn (B.Z.); liumjo@163.com (M.L.); wl_19830807@sohu.com (L.W.)

**Keywords:** heavy metal stress, remote sensing, time series, WOFOST, EEMD, trend component

## Abstract

The use of remote sensing technology to diagnose heavy metal stress in crops is of great significance for environmental protection and food security. However, in the natural farmland ecosystem, various stressors could have a similar influence on crop growth, therefore making heavy metal stress difficult to identify accurately, so this is still not a well resolved scientific problem and a hot topic in the field of agricultural remote sensing. This study proposes a method that uses Ensemble Empirical Mode Decomposition (EEMD) to obtain the heavy metal stress signal features on a long time scale. The method operates based on the Leaf Area Index (LAI) simulated by the Enhanced World Food Studies (WOFOST) model, assimilated with remotely sensed data. The following results were obtained: (i) the use of EEMD was effective in the extraction of heavy metal stress signals by eliminating the intra-annual and annual components; (ii) LAI_df_ (The first derivative of the sum of the interannual component and residual) can preferably reflect the stable feature responses to rice heavy metal stress. LAI_df_ showed stability with an R^2^ of greater than 0.9 in three growing stages, and the stability is optimal in June. This study combines the spectral characteristics of the stress effect with the time characteristics, and confirms the potential of long-term remotely sensed data for improving the accuracy of crop heavy metal stress identification.

## 1. Introduction

Heavy metal pollution in farmland not only destroys the normal function of the soil and hinders crop growth, but also endangers human health through the food chain [1,2,3]. According to relevant statistical information, the amount of contaminated grains is up to 12 million tons in China every year [4]. Because of the serious threat to the quality of agricultural products and the sustainable development of agriculture, accurate and rapid monitoring of heavy metal pollution has become a topic of critical importance [5,6].

The use of satellite remote sensing data to monitor a wide range of heavy metal pollution in farmland has become an important technical method [7]. The current monitoring of heavy metal pollution stress in crops using remote sensing technology is based mainly on the physiological and biochemical characteristics induced by heavy metal stress and the corresponding spectral response [8,9,10,11]. For example, some experiments have revealed that heavy metal stress leads to decreases in the chlorophyll content and reflectivity in the near infrared wavelength bands, an increase in the reflectivity in the red wavelength bands, and a red edge blue-shift [12,13,14]. However, the growth mechanism of crops under external stress is complex, and other environmental stress factors (e.g., pests and diseases, nutrient stress, and water stress) may have the same effect on crops [15], resulting in similar spectral variation characteristics. These limitations are larger when identifying heavy metal stress based only on stress spectral response parameters. For instance, a sensitive method to extract heavy metal stress from multifarious environment stressors is essential for exploring the features that can truly characterize crop heavy metal stress.

Various factors may cause stress on crop growth in the complex farmland ecosystem. These environmental stress factors can be divided into volatile factors (such as pests and diseases, moisture, climate anomalies, and nutritional stress) and stable factors (such as heavy metal stress and salt stress) for crops [16]. For a given region, stable factors persist for a certain period of time, and it is uncertain whether a volatile factor exists or not. The characteristics of heavy metal contaminants include poor migration ability, degradation resistance, and irreversibility in the soil [17]. Thus, heavy metal stress exists during the entire crop growth cycle and has a consistent long-term influence on growth. The effect of heavy metal stress on crop growth in different years is thus similar. Other stressors are affected by diverse factors such as climate, planting habit, and management conditions [18] with distinct interannual changes often existing in one or several growth cycles of crops. These stressors show volatility and are short-acting within the year and on interannual cycles. For example, rice stripe disease and rice planthopper damage occur in the seedling and booting stages, respectively [19,20]. In light of the stable interannual variation of the influence of heavy metal stress on crops, heavy metal stress can be effectively distinguished from other stressors by continuous monitoring of crops on a long time scale.

Ecophysiological changes of crops under environmental stress can be characterized by damage to a series of photosynthetic apparatuses [21,22]. Leaf Area Index (LAI) is a key physical quantity that characterizes the growth status of vegetation and is an indispensable parameter to describe plant photosynthesis and material energy exchange. Previous research has employed LAI to indicate the morphological and color changes of crops and to monitor the heavy metal stress of crops through the band response characteristics of visible light to shortwave infrared [23,24,25,26]. Therefore, LAI can be used as a valid indicator for monitoring the crop stress. 

Currently, it is difficult to use remote sensing technology to directly estimate the LAI with long time resolution. The World Food Studies (WOFOST) model can achieve the dynamic simulation of LAI with a one-day time step through assimilating with remotely sensed information [4,27,28]. When the physiological functions are at the optimal state, the crop growth curve of LAI can be simulated by potential growth level under non-stress conditions [15]. Crops experience environment stress if suboptimal growth conditions cause their crop physiological functions to decline from their physiological standard [29], and the amplitude of the LAI signal will be dampened once the crops suffer stress. The total stress information is hidden in the reduced amplitude, including heavy metal stress and other stress information. In consequence, the amplitude of the LAI signal on stressed rice is the subtraction of the intrinsic growth signal of the crop and total stress signal. The LAI time series can thus be considered to contain three types of information: the inherent growth tendency, persistent environmental stress (heavy metal stress), and other stressors.

Thus, in this study, we extracted the rice heavy metal stress signal feature by using a decade of remotely sensed data. By focusing on the difference in stress effects at different time scales for various environmental stress types, the LAI time series simulated by the enhanced crop growth model (WOFOST) was decomposed to three kinds of signals through Ensemble empirical mode decomposition (EEMD). After getting rid of some other stress and crop innate growth signals, the remaining signal containing heavy metal stress information was extracted. Finally, we explored the features that can characterize the heavy metal stress accurately and evaluated its stability. These three analyses are the main objectives of this paper. This paper provides a reliable method using long time-series remote sensing data for accurate identification of heavy metal stress in crops, which could be further applied in the precise monitoring of heavy metal stress.

## 2. Study Area and Data

### 2.1. Study Area

**Figure 1:**
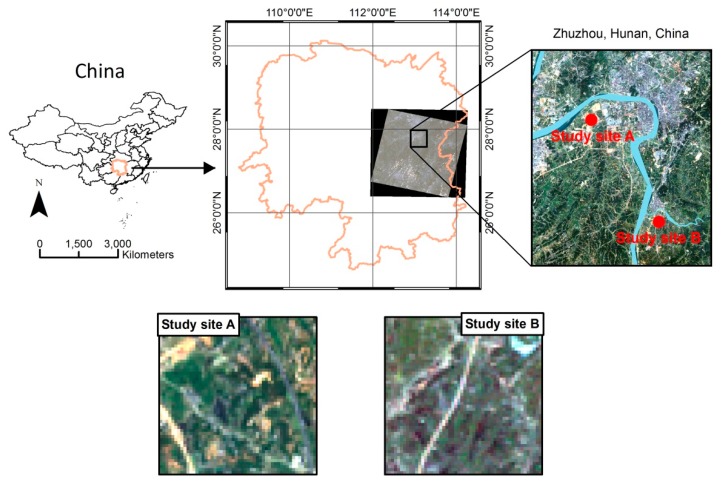
Study area in Zhuzhou, Hunan province, China, showing the locations of the two field sites under severe pollution level (site A) and moderate pollution level (site B).

The study area is located in northern Zhuzhou (Hunan Province, China). The area is located in a humid subtropical monsoon climate zone with sufficient light intensity and with an annual average temperature of 15.5 °C–25 °C. The annual precipitation is 1250–1500 mm. This province hosts non-ferrous metals; rare metal mining and smelting operations are concentrated in the Xiangjiang River Basin. In addition, a significant amount of industrial waste water is discharged into the surrounding environment, causing severe heavy metal contamination of water and the soil. Two experimental sites adjacent to the Xiangjiang River, labeled A and B in Figure 1, were selected for study. On the basis of soil sample analysis, the average contents of Cd, Hg, and Pb in the soil were greater than the corresponding background value for soil heavy metal concentration. And therefore, the concentrations of main heavy metals including Cd, Hg and Pb at these two sites are shown in Table 1. The soil and the stress rates in sites A and B were determined as high and medium levels of heavy metal stress, respectively.

### 2.2. Data Preparation

The climatic conditions of Zhuzhou City during the crop growing period include a lot of cloudiness, rain, and fog, thus, the use of a single remote sensing data source will result in abundant missing data. Previous research reported that although Landsat 5 and Landsat 7 differ in calibration accuracy, the response rate difference of TM (Thematic Mapper) and ETM+ (Enhanced Thematic Mapper Plus) is less than 0.02 [30]. The study [31] has concluded that the two sensors of Landsat 7 and Landsat 8 can be used together under normal circumstances because of their similarities, which shows the feasibility of using combined data to analyze time-series information even though some differences exist between them. Therefore, images of Landsat 5 TM, Landsat 7 ETM+, and Landsat 8 OLI (Operational Land Imager) complement each other. All cloudless Landsat images for the entire growth season of June to September during 2007–2016 were obtained from the United States Geological Survey (http://glovis.usgs.gov/). These were used to retrieve the Normalized Difference Vegetation Index (NDVI), which is the best indicator of vegetation growth status and vegetation coverage. In 2010, however, cloud pollution and restricted space coverage resulted in few effective cloud-free Landsat images in the study area, multi-sensor observations of the HJ-1 CCD and Landsat 8 OLI have been used to compensate for the spatial deletion of LAI synthetic products [32]. We thus acquired HJ-1 CCD data from the Resource Satellite Center website (http://www.cresda.com/CN/) to supplement the missing images. Table 2 shows the situation of selected images at sites A and B for a 10-year period. The parameters of the satellite sensors which were employed are shown in Table 3. Considering the differences in radiation performance and band setting, spatial position, atmospheric condition, and other factors, we preformed preprocessing work such as cross-radiation calibration among sensors and geometric and atmospheric correction. The atmospheric correction of the images was processed by the FLAASH (Fast Line-of-sight Atmospheric Analysis of Hypercubes) modeling tool in the ENVI (The Environment for Visualizing Images) software (Exelis Visual Information Solutions, United States). Geometric correction of the Landsat 5, Landsat 8, and CCD images was completed using the stratified registration method based on the Landsat 7 image captured on 31 July 2007. This was conducted with an RMSE (Root Mean Square Error) of 0.5 pixels to eliminate registration errors. To be consistent with the Landsat data, CCD time series images were re-projected to the UTM (Universal Transverse Mercator)-WGS84 projection. Such measures are prerequisites for efficient use of long-term, multi-sensor remotely sensed data.

The meteorological data applied in this study includes daily maximum and minimum air temperature and the daily actual hours of sunshine. The data for 2007 to 2013 were acquired from Zhuzhou weather station, and those for 2014 to 2016 were downloaded from the China Meteorological Data Sharing Service System. These data were transformed into solar radiation as a climatic input parameter for the enhanced WOFOST model [33,34].

## 3. Methods

### 3.1. Simulating LAI Time Series by the Enhanced WOFOST Assimilated with Remotely Sensed Data

**Figure 2:**
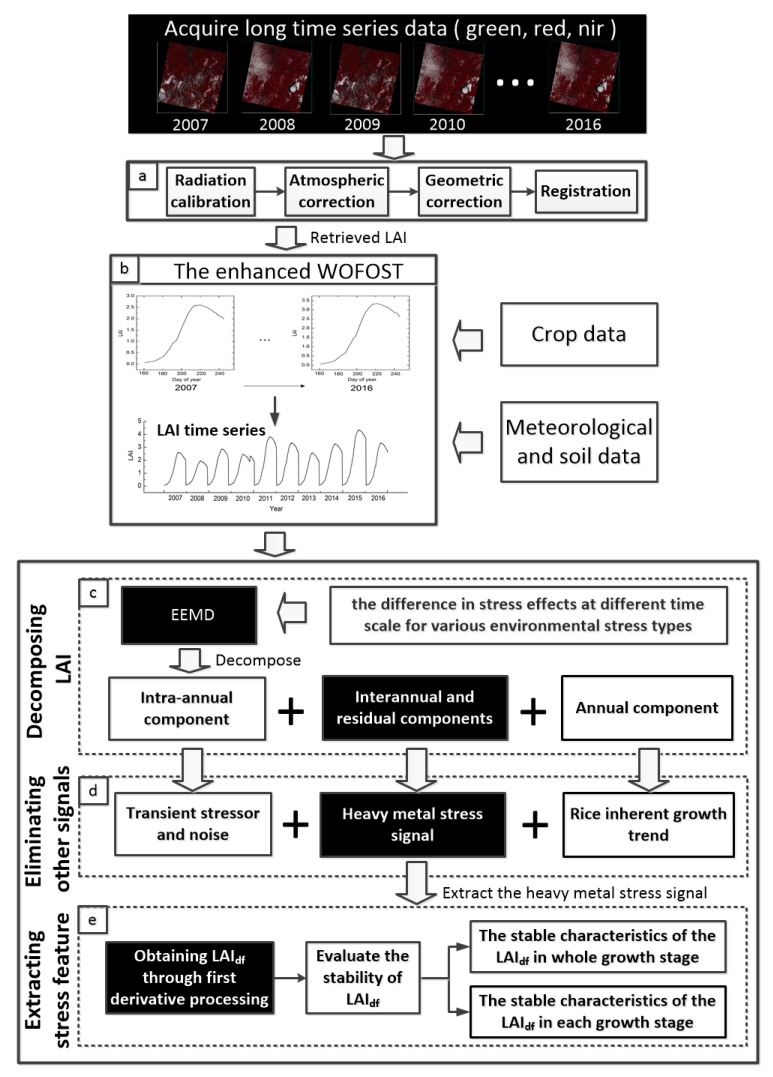
Technical flow chart of extraction of rice heavy metal stress signal features based on long time series LAI using EEMD. LAI: Leaf Area Index; LAI_df_: The first derivative of the sum of the interannual component and residual; EEMD: Ensemble Empirical Mode Decomposition.

In order to convert discontinuous remote sensing information into time-continuous crop information, the assimilation technique and the enhanced WOFOST model were combined to dynamically monitor the time continuity of the growth parameters. The WOFOST model is a process-based quantitative analysis model which can obtain the continuous growth parameters of the stressed crops. To do so, it quantitatively simulates the distribution of roots, stems, leaves, and storage organs (seeds), in addition to the daily growth status of crops in the growing process [4,27,35]. Two important indices corresponding to physiological function variations, including photosynthesis and dry-matter formation, were incorporated into the enhanced WOFOST model [15]. The input parameters of the enhanced WOFOST model were climate, soil, and crop data. In addition to the data previously obtained, the LAI was also used for assimilation in calibrating the stress factors. The empirical model based on actually measured data and spectra have been validated effectively to invert LAI using NDVI [4], with the following formula:

y = 0.3629e^3.075x^, (1)

where x is the value of NDVI and y is the value of LAI. We conducted a supervised classification of the images of two plots in each phase and retrieved the NDVI for paddy field pixels (Figure 2a). The input LAI was calculated from the average NDVI value of rice pixels at two sites. The enhanced WOFOST model was then used to simulate the decadal (2007–2016) rice stress response parameters LAI. The long-term series curve of LAI was then obtained on a time scale, after integrating the decade of simulation results (Figure 2b).

### 3.2. Ensemble Empirical Mode Decomposition

To extract the heavy metal stress signal in our experiment, we used Ensemble Empirical Mode Decomposition (EEMD, Figure 2c), which is a method of signal analysis similar to Fourier transform and Wavelet transform. Based on Empirical Mode Decomposition (EMD) introduced by Wu and Huang [36], EEMD is a noise-assisted data analysis method that is able to effectively analyze nonlinear and non-stationary signals. The EMD time-frequency analysis method was proposed by Huang in 1998 [37]. The signal decomposed by EMD generates modal aliasing when a jump occurs in the time scale. This led to the development of EEMD, which adds white noise to the signal to be decomposed and utilizes the characteristics of the zero mean of Gaussian white noise to effectively solve the aliasing phenomenon and retain the real signal [36,38]. In the EEMD method, fluctuation and trend information contained in the original data on different time scales is expressed as the sum of Intrinsic Mode Function (IMF) satisfying the Hilbert transform requirement and the residual function [37]. Each IMF represents information from the original data on different scales, and the residual signal represents the trend. EEMD can also be applied to represent the signal density and intensity in frequency; in this study, however, we focus mainly on the variation trend at different time scales. The advantage of EEMD is that it does not behave like Fourier analysis and wavelet analysis, which rely on the selection of the basis function. Rather, each of its resolution parameters can impersonally determine its meaning, and we can obtain the selected constituent.

There are studies illustrating that the LAI time series can be decomposed into three modes and a residual of different scales by means of EEMD, including intra-annual, annual, and inter-annual [39,40,41]. The intra-annual mode with high frequency has high time resolution and low frequency resolution, which reflects the details of the signal and can detect a transient phenomenon entrained in the signal. This anomaly is related to the volatility and shortness of other environmental stressors, so other environmental stressors are contained in the high frequency components of the signal, whereas the high frequency component contains not only the anomaly information but also the noise information induced by various environmental factors. The crucial characteristic of the inherent trend of crop growth is that it has a regular cycle. For the parameter of crop growth, there are usually oscillations with one peak in a year. Thus, we considered the annual mode with a one-year period stable cycle as the inherent trend. In addition to intra-annual and annual modes, heavy metal stress information was included in the remaining signal (Figure 2d).

**Figure 3:**
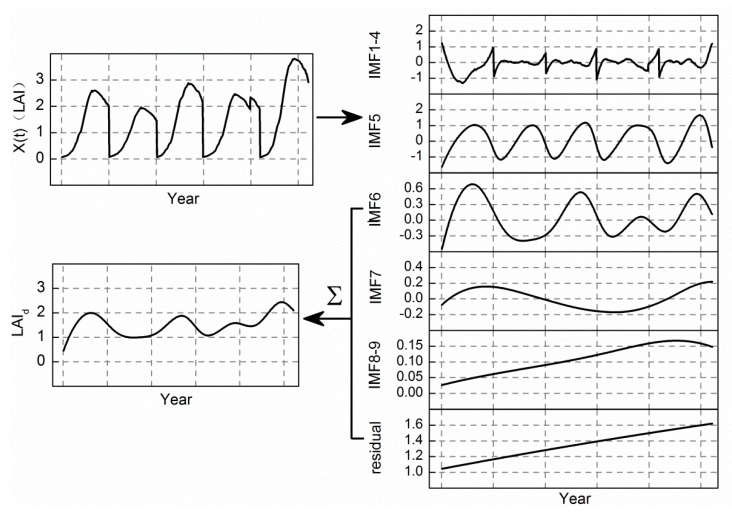
Rationale for extracting heavy metal stress signal based on ensemble empirical mode decomposition (EEMD). The LAI time series is broken down into nine modes and a residual that are divided into three main components: other short-term stress (IMF1-4), the crop inherent growth trend (IMF5), and heavy metal stress (IMF6-residual). LAI: Leaf Area Index; IMF: Intrinsic Mode Function.

On the basis of the above, we introduced the intra-annual mode (IMF1-4) on behalf of some short-term stress. The steady change observed every year after removing the noise is the inherent trend, i.e., the annual mode (IMF5). The inter-annual mode and residual contain heavy metal stress (LAI_d_) (Figure 3). Therefore, we used EEMD to acquire the components of the signal containing heavy metal stress information to enable further extraction of the heavy metal stress feature.

### 3.3. Extracting Stable Features of the Rice Heavy Metal Stress

Derivative spectral analysis is a method which can be used to reduce the influence of background noise such as that from the soil. This improves the signal-to-noise ratio and the correlation between the enhanced spectral parameters and biochemical composition [42,43,44]. Some studies have derived the vegetation index, and other signs of vegetation attribute data, to extract the signal of sudden changes in the vegetation [45,46]. The first derivative of the NDVI time curve indicates the rate of change of the phenology [47]. The IMF is related to the frequency of the spectral reflectance [41], and the other noise information in the isolated heavy metal stress information is relatively gentle when compared to the change in stress information. Therefore, we believe that the use of derivative technology can further eliminate the influence of the low-frequency components and remove the interference from other backgrounds to more effectively preserve the characteristics of heavy metal stress. The time series curves of LAI_d_ were derived to show the slope of the curve, i.e., its rate of change (Figure 2e) using the following formula:

(2) $$LAI_{df}=\frac{LAI_{d}{}_{t+1}-LAI_{d}{}_{t}}{\Delta DOY}$$

where the value of $LAI_{df}$ is the first derivative of $\;LAI_{d}$, $LAI_{d}{}_{t}$ is the value at previous phase and $LAI_{d}{}_{t+1}$ is the value at later phase, ΔDOY is the interval between the previous phase and later phase, here ΔDOY is 1 as the daily LAI can be simulated by the enhanced WOFOST. 

To quantify the performance of signal stability, the similarity between the annual signal curves was evaluated with the coefficient of determination, which was computed by:

(3) $$R^{2}=\frac{{\left[\sum_{i=1}^{N}\left(y_{ai}-\bar{y_{a}}\right)\sum_{i=1}^{N}\left(y_{bi}-\bar{y_{b}}\right)\right]}^{2}}{\sum_{i=1}^{N}{\left(y_{ai}-\bar{y_{a}}\right)}^{2}\sum_{i=1}^{N}\left(y_{bi}-\bar{y_{b}}\right)}$$

where $y_{ai}$ and $y_{bi}$ are the values of the two signals being compared, $\bar{y_{a}}$ and $\bar{y_{b}}$ are the average values of the two signals. N is the number of days. R^2^ ranges from 0 to 1. The higher the R^2^ value is, the more similar the signal curves, and the better the stability of the signal.

## 4. Results

### 4.1. Decomposition of LAI Time Series

On the basis of EEMD, the LAI time series was decomposed into three main components—intra-annual, annual, and interannual—as well as a residual. The standard deviation of the Gaussian white noise added in the EEMD (Nstd) and the number of times the noise was added (NE) were used to determine the decomposition effect. The Nstd range is 0.01–0.4; we chose increments of 0.01, 0.05, 0.1, 0.2, and 0.3 for adding NE 50 and 100 times. The experiment revealed that regardless of the number of additions when Nstd was the same, the decomposition results were similar. Therefore, we set NE to 100 times.

Because the annual trend of crop growth is essentially constant for ten years, the experiment was conducted using several prepared Nstd values according to a benchmark of the coefficient of determination (R^2^) of the annual component among the ten years. The value with the highest correlation was chosen as the Nstd used in this study. Table 4 illustrates the coefficient of determination of the annual component from year to year after setting up the different parameters. When the value of Nstd was 0.3, the average R^2^ reached 0.927, and the relationship was at its closest. What’s more, the *p* value was less than 0.05 and the correlation was prominent. Therefore, 0.3 was selected as the most appropriate Nstd value for our decomposition.

**Figure 4:**
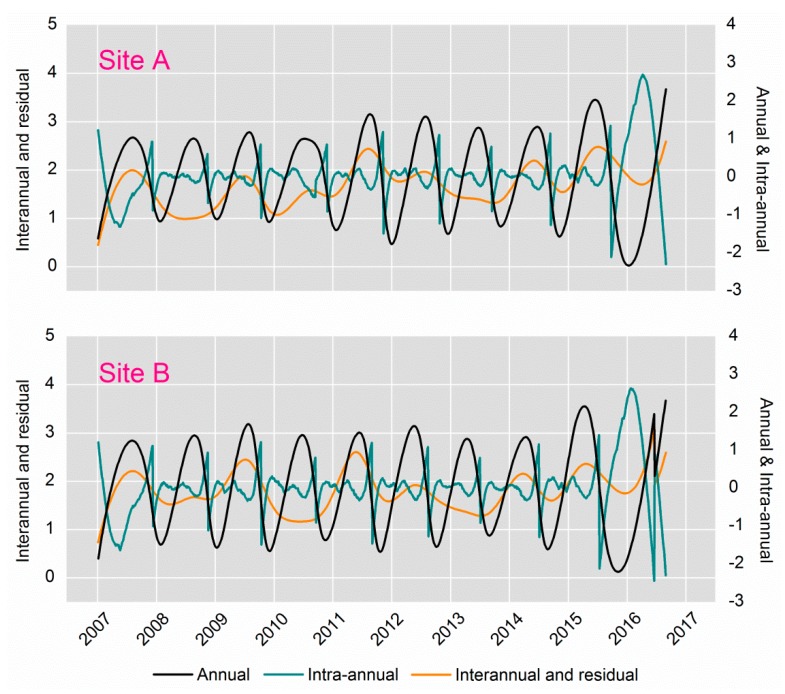
The LAI time series decomposed into three main segments by means of EEMD at both sites A and B. The three segments are intra-annual, annual, interannual plus residual, respectively. LAI: Leaf Area Index; EEMD: Ensemble Empirical Mode Decomposition.

After EEMD decomposition the LAI time series produced ten (IMF1-9 and a residual) compositions, respectively. According to the analysis in the method, three kinds of signals were extracted; the signal of IMF1-4 in the LAI time series with high fluctuation frequency, the changes during the year, and interannual changes which were highly irregular. The intra-annual component is the green line in Figure 4. The signal of IMF5 in the LAI time series is the annual component, the variation trend of which is one cycle per year (black line in Figure 4).

The rest of the signals are the interannual component and residual (orange line in Figure 4). In this way, the acquisition of the three kinds of signals helps us to peel off the other stress information and separate heavy metal stress signals.

### 4.2. Elimination of Transient Stressors and Noise

**Figure 5:**
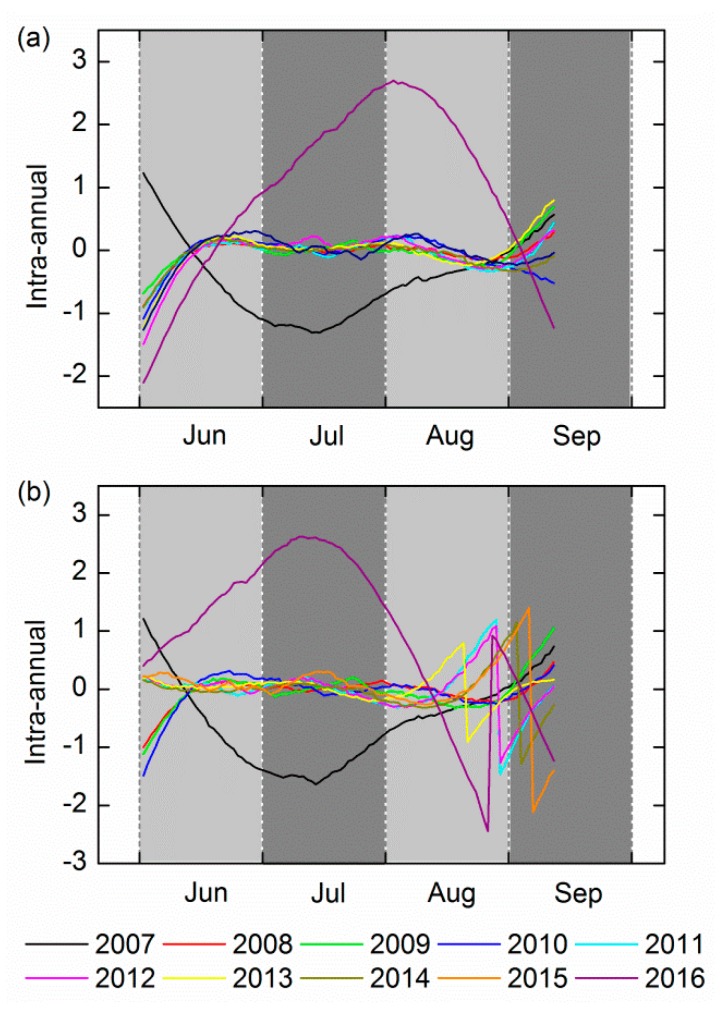
The dynamics of the 10-year intra-annual component for the entire growing period at (**a**) site A and (**b**) site B.

The intra-annual component for the entire growth period of the decade is shown in Figure 5, with the exception of 2007 and 2016, the signal for 2008–2015 fluctuated frequently throughout the whole growing process. Table 5 lists the R^2^ values of the signal across the ten year period for each growth stage (June (tillering stage), July (booting stage), August (flowering stage), and September (ripening stage)). The range of average R^2^ values was from 0.268 to 0.360. The lower R^2^ demonstrated that they had low similarity, and thus that stability was not strong.

The noise from other environmental factors that were present can cause long-term fluctuations in the signal, so the fluctuations for the years from 2008 to 2015 may be the result of noise. Conversely in 2007 and 2015 the signal frequency is lower compared to other years, and the vibration amplitude is larger. This probably results from transient stress, such as moisture, pests, and diseases, because the impact on rice from these factors relative to the environmental factors is more obvious. The conclusions drawn from the analysis are that the intra-annual component can be proven to be unstable signals containing transient stressors and noise.

### 4.3. Exclusion of the Inherent Growth Trend of Rice

**Figure 6:**
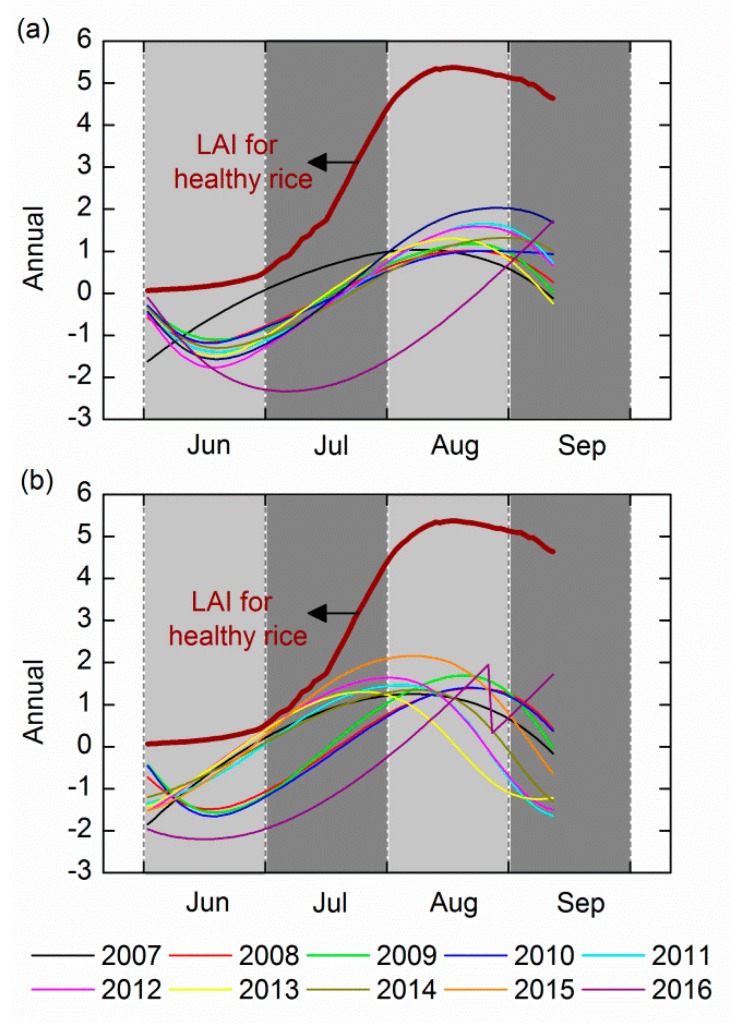
The comparison of the dynamics of the 10-years annual component in the entire growing period and the LAI time series on healthy rice at (**a**) site A and (**b**) site B. LAI: Leaf Area Index.

Figure 6 displays the contrast of the annual component and the LAI time series for healthy rice simulated with the enhanced WOFOST. In general, there were oscillations with a peak in the whole growth period, and their trajectories were characterized by similarity. Table 6 shows the R^2^ between LAI time series for healthy rice and the annual signal at both sites A and B. At site A, despite the abnormal trend in the years of 2016 and 2007, the average R^2^ reached 0.853; the average R^2^ at site B was 0.754 and the R^2^ in 2016 is also not high enough. In general, the LAI time series on healthy rice is relatively highly correlated with the annual signal. The results verified that the annual component was able to reveal the signal response to the rice’s inherent growth trend.

### 4.4. Acquisition of Stable Features of Rice Heavy Metal Stress

**Figure 7:**
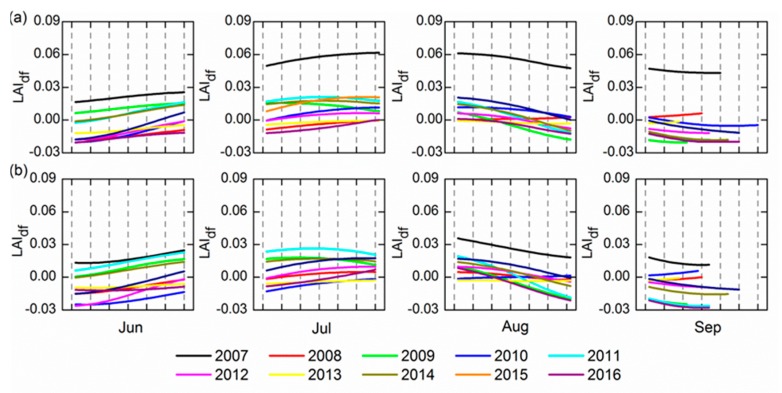
Trends of 10-year LAI_df_ at (**a**) site A and (**b**) site B in the four growing stages. LAI_df_: The first derivative of the sum of the interannual component and residual.

By eliminating the above two kinds of components, the heavy metal stress information remains in the sum of the inter-annual component and the residual (LAI_d_). To further exclude the interference from other low frequency signals and extract the stable heavy metal stress feature, the LAI_df_ time series was obtained through the first derivative processing to LAI_d_. These LAI_df_ were compared in the four growing periods (Figure 7). The growing trend in June and July and the reducing trend in August and September were relatively stable during the 10 years of change. Nevertheless, in 2011 and 2014 there were abnormal change trends of LAI_df_ showed by both sites A and B in July.

We conducted a stability analysis from two aspects. On the one hand, the analysis of the R^2^ values for the LAI_df_ signal in each growth period across the ten years is shown in Table 7. We can see that the LAI_df_ signal has high similarity apart from in July; the similarity is at its best in June at both sites A and B and the stability is relatively strong. On the other hand, because the LAI_df_ sequences were close to the linear trend in the four growth periods each year, their linear fitting was used to analyze their trend discrepancy. The fitting precision in addition to some of the abnormal values were found to be relatively high by experiment, indicating that this method more reliably denotes the dynamic. The slope ratios of each line fitting equation (LAI_dfs_) reflect the dynamic situation of the LAI_df_. Table 8 compares the standard error of 10-year LAI_dfs_ in different growth periods.

The results show the fluctuant magnitude of the LAI_dfs_ across the 10 years. The fluctuations of the LAI_dfs_ for the decade were both minimal in June regardless of the site. In summary, it is concluded that the stability of trend of the LAI_df_ was most obvious in June. It is possible that the LAI_df_ was less sensitive in other periods, and the stability was thus not prominent.

**Figure 8:**
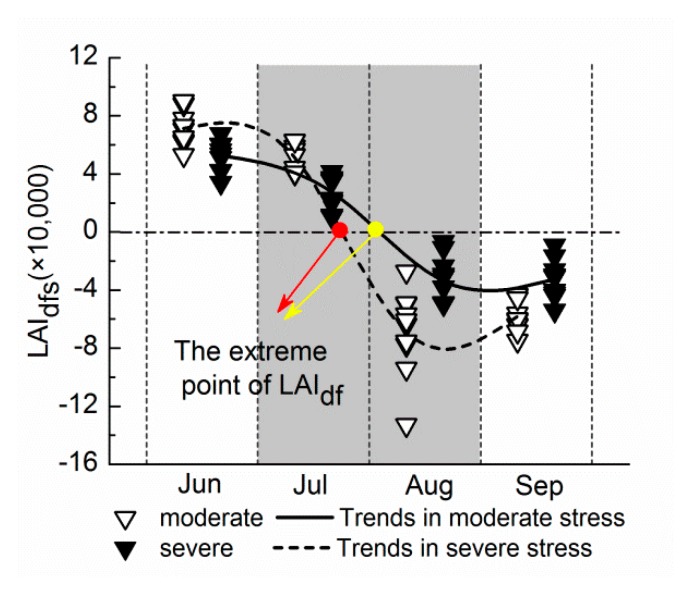
The LAI_dfs_ under two different levels of heavy metal pollution in every growing process. The curves represent the connection of the mean for multi-year LAI_dfs_ in the four months, reflecting the overall variation trend in the entire growth period. LAI_dfs_: The slope ratios of the line fitting equation of LAI_d_.

The dynamics of LAI_dfs_ for sites A and B over the entire growth phase are shown in Figure 8. The LAI_dfs_ at site A reached the zero point in August, and site B reached zero in July. The results demonstrate that the overall dynamic of LAI_df_ throughout the growing season at site A was similar to that at site B: the trajectories appeared to grow first and then fall as a whole, and all showed a peak during July to August. Based on the above analysis, the LAI_df_ of ten years has an equivalent variation trend both in the whole growth period and in each of the growing stages. This indicates that LAI_df_ can be regarded as a stable feature to represent the rice heavy metal stress signal.

## 5. Discussion

The EEMD method for extracting the heavy metal stress signals in rice based on long time series LAI was presented. The reasonable characteristics of heavy metal stress were produced using a first derivative operation. The LAI_df_ had a good stability in the four growth periods and for the full growth process. This was supported by the coefficient of determination, the standard deviation statistic and trajectory analysis for LAI_df_, which indicated that the EEMD is a reliable means to extract the heavy metal stress signal.

The peak of the LAI_df_ indicates that the change rate of LAI_d_ is maximal at this time only under heavy metal stress. LAI_d_ can be regarded as the reduced magnitude of the curve of the LAI time series in rice under heavy metal stress relative to non-stressed rice. In other words, the effect of stress on the crop growth. During the vigorous vegetative growth period, the growth center is the growth and development of vegetative organs. Studies have shown that heavy metal pollution in this period can cause serious damage to the chloroplast such as the dissolution of the membrane system of the chloroplast [48,49,50,51,52]. Thus, the inhibitory effect on rice growth induced by heavy metal stress will get faster. Later, owing to the rapid increase in crop biomass, the heavy metal content in the crop will be subjected to a large degree of dilution, and the performance of heavy metal stress begins to weaken. Therefore, it is possible that during July to August, the maximum inhibition of photosynthesis was reached during the vigorous growth period; the change rate of the effect of heavy metal stress at this stage reached the maximum; and LAI_df_ also reached its maximum. 

It could be argued that in contrast to the trajectory of LAI_df_ over the ten years, there are two years with abnormal readings in July. One reason for this is that, relative to the other three months, this period does not have a very clear stable trend. The trend in this stage also exhibits greater uncertainties. Moreover, the trend inconsistencies are most likely to result from sensor inconsistencies (e.g., orbital drift or defective corrections) or between-sensor inconsistencies (e.g., historic processing differences or differences in sun-target-sensor geometry) [39]. The combination of multi-source sensor data has an effect on the retrieved LAI, which as the input parameter of the enhanced WOFOST; ultimately has influence on the simulation results of the LAI. Another reason may be that the LAI_df_ is not stable enough on the scale of the month; this can be further verified by selecting other indicators, which in the case of other conditions remain unchanged. All of these factors contribute to the broader uncertainties associated with the LAI_df_. In general, even though the performance of LAI_df_ in July is not particularly desirable, the trend of change over the 10 years was relatively stable. The LAI_df_ can still be used as a stable feature to reveal the rice heavy metal stress.

This study is based on the discrepancy between the stress features at different time scales; thus, we accessed the dataset from 2007–2016. The land use type of the study area during the 10 years showed little difference and the heavy metal stress in the study area is dominant status because of the potential hazards. Therefore, the extent of crop stress by heavy metals can be regarded as a constant factor. However, the retrieved LAI was calculated from the obtained rice pixels through supervised classification in the region, which can produce some error. In addition, the LAI values of the study area were averaged at each phase, and the number of rice samples in the past decade changed, which could have also had an effect on the results. The influencing factors of the enhanced WOFOST assimilation model are weather parameters and crop parameters. Our 10 years of meteorological data were obtained from a weather station. Owing to the small range of the study area, the variation in weather conditions did not lead to uncertainty, however, it is necessary to consider the regional distribution of climate data if this method is to be applied on a large regional scale [15]. For the EEMD decomposition model, different parameters affected the results of the decomposition, therefore, uncertainty exists in the screening process. Moreover, if the origin signal is not properly de-noised beforehand, then the noise involved in decomposition increases the number of layers of decomposition. Unnecessary decomposition layers cause error and cumulative errors, which reduces the accuracy of signal decomposition [53]. However, the choice of different de-noising methods before the signal is input will produce effects of varying degrees for the original signal, and the results of the decomposition will also vary in performance. The LAI time series was not properly de-noised prior to decomposition in our experiment. Although these errors and uncertainties exist, it does not mean that the features we acquired from the experiment are not exact. The characteristics of the heavy metal stress signal we obtained are, to some extent, stable.

For the obtained rice heavy metal stress signal features, the stability is still insufficient. We can further explore whether other indicators can better characterize heavy metal stress based on the proposed method. We can establish a comprehensive index of some indicators to improve the stability; and more accurately assess the rice characterization results from the differences in stress levels. The determination of a comprehensive evaluation index and the establishment of an evaluation model of heavy metal stress are topics for our future research. 

## 6. Conclusions

This study describes a new concept of extracting stable heavy metal stress features and provides a research method for accurate remote sensing identification of heavy metal stress in crops. In this paper, we used the EEMD approach to extract rice heavy metal stress signals based on multi-year remote sensing data and excavated the varying features of heavy metal stress signals. The change trends of the LAI_df_ that characterize the heavy metal stress in the whole growth period were similar, and were also consistent in each growth period. The stability of the LAI_df_ response to rice heavy metal stress can be best evaluated in June.

In summary, according to differences in temporal characteristics presented by all types of stress effects, the method of EEMD can effectively be used to identify rice heavy metal stress. The LAI_df_ manifests as a stable heavy metal stress feature and well embodies the desired meaning: the influence of heavy metal stress on rice growth. This study provides a scientific basis for remote sensing monitoring of crop environmental stress. The EEMD method combined with the stress mechanism, stress parameters, and stress time effects can promote the accuracy of crop heavy metal stress monitoring.

## Figures and Tables

**Table 1:** Heavy metal concentration (unit: mg/kg) in the surface soil of the two study sites.

Study Site	Background Value ^a^	Hg	Pb	Cd	Pollution Level
		0.20	82.78	1.43	
A (27°49′ N,113°02′ E)	Soil	0.51	109.93	3.27	Severe
	Exceed rate ^b^	155.00%	32.80%	128.67%	
B (27°39′ N,113°09′ E)	Soil	0.29	89.67	2.25	Moderate
	Exceed rate	45.00%	8.32%	57.34%	

Note: ^a^ is the heavy metal background values, based on data from the Hunan Institute of Geophysical and Geochemical Exploration in China [4,15]; ^b^ is the exceed rate, ^b^ = (Soil value-Background value)/Background value.

**Table 2:** Overview of the data source, image acquisition dates, and number of scenes from 2007 to 2016 at site A and site B.

**Data Source**		**2007**	**2008**	**2009**	**2010**	**2011**
		**DOY**	**DOY**	**DOY**	**DOY**	**DOY**
Landsat 5	A	212, 260	263	169, 201, 233, 249		159, 207, 271
	B	212, 260	199, 263	201, 233, 249		159, 207, 271
Landsat 7	A	220	159, 223	241	260	183, 231
	B	220	207, 223	193, 241	260	183, 231
Landsat 8	A					
	B					
HJ-1	A				181, 185, 224, 234	
	B				181, 185, 224, 234	
**Data Source**		**2012**	**2013**	**2014**	**2015**	**2016**
		**DOY**	**DOY**	**DOY**	**DOY**	**DOY**
Landsat 5	A					
	B					
Landsat 7	A	170, 186, 250	172, 220, 252	191, 255	178, 194, 210	213
	B	170, 186, 250	172, 220, 252	191, 255	178, 194, 210	213
Landsat 8	A		212	247	218	157, 205, 269
	B		212	247	218	205, 269
HJ-1	A					
	B					

DOY: Day Of Year.

**Table 3:** General conditions for the sensors of Landsat 5, Landsat 7, Landsat 8, and HJ-1 satellites.

Sensors	Spectral Band (µm)				Revisit Cycle (day)	Resolution (m)	Scanning Width (km)
	Blue	Green	Red	Near-Infrared			
TM	Band1: 0.45–0.52	Band2: 0.52–0.60	Band3: 0.63–0.69	Band4: 0.76–0.90	16	30	185
ETM+	Band1: 0.45–0.52	Band2: 0.52–0.60	Band3: 0.63–0.69	Band4: 0.77–0.90	16	30	185
OLI	Band2: 0.45–0.51	Band3: 0.53–0.59	Band4: 0.64–0.67	Band5: 0.85–0.88	16	30	185
CCD	Band1: 0.43–0.52	Band2: 0.52–0.60	Band3: 0.63–0.69	Band4: 0.76–0.90	2	30	711

TM: Thematic Mapper; ETM+: Enhanced Thematic Mapper Plus; OLI: Operational Land Imager; CCD: Charge-Coupled Device.

**Table 4:** The coefficient of determination (R^2^) of the 10-years inherent growth trend decomposed by EEMD (Ensemble Empirical Mode Decomposition) set different parameters.

Nstd (NE = 100)	0.01	0.05	0.1	0.2	0.3
2007–2008	0.880	0.288	0.517	0.896	0.954
2008–2009	0.957	0.978	0.980	0.990	0.981
2009–2010	0.063	0.806	0.908	0.939	0.943
2010–2011	0.035	0.885	0.910	0.887	0.879
2011–2012	0.221	0.997	0.989	0.976	0.980
2012–2013	0.469	0.938	0.943	0.917	0.937
2013–2014	0.968	0.882	0.858	0.742	0.780
2014–2015	0.591	0.932	0.983	0.997	0.995
2015–2016	*	*	0.614	0.989	0.895
Mean value	0.532	0.838	0.856	0.926	0.927

The analysis is based on *p* < 0.05. * is the eliminated data where *p* > 0.05 and the correlation is quite low. Nstd: The standard deviation of the Gaussian white noise added in the EEMD; NE: The number of times the noise was added in the EEMD.

**Table 5:** R^2^ and mean between the adjacent two years for the intra-annual signal at both sites A and B.

Year	R^2^_Site A				R^2^_Site B			
	June	July	August	September	June	July	August	September
2007–2008	0.188	−0.035	0.227	0.275	0.102	−0.030	0.157	0.140
2008–2009	0.240	0.167	0.329	0.382	0.696	0.416	0.411	0.268
2009–2010	0.492	0.402	0.379	0.249	0.396	0.633	0.528	0.370
2010–2011	0.395	0.823	0.653	0.419	0.494	0.552	0.194	0.328
2011–2012	0.296	0.093	0.279	0.462	0.271	0.233	0.295	0.198
2012–2013	0.298	−0.036	0.180	0.393	0.340	0.233	0.030	0.585
2013–2014	0.593	0.697	0.315	0.390	0.264	0.477	0.143	0.216
2014–2015	0.370	0.457	0.513	0.518	0.313	0.346	0.692	0.579
2015–2016	0.194	0.051	0.177	0.154	0.193	0.079	−0.034	−0.083
Mean	0.340	0.291	0.339	0.360	0.341	0.327	0.268	0.289

**Table 6:** Statistic of the R^2^ between the LAI (Leaf Area Index) signal on healthy rice and the annual signal at both sites A and B.

Study Site	2007	2008	2009	2010	2011	2012	2013	2014	2015	2016	Mean
A	0.588	0.962	0.934	0.96	0.966	0.962	0.881	0.951	0.964	0.365	0.853
B	0.731	0.971	0.92	0.954	0.613	0.668	0.725	0.958	0.68	0.316	0.754

**Table 7:** R^2^ and mean between the adjacent two years for the LAI_d_ signal at both sites A and B.

Year	R^2^_Site A				R^2^_Site B			
	June	July	August	September	June	July	August	September
2007–2008	0.946	0.986	0.891	0.970	0.946	0.980	0.986	0.796
2008–2009	0.946	0.794	0.934	0.912	0.978	0.377	0.993	0.644
2009–2010	0.945	0.693	0.895	0.955	0.930	0.521	0.959	0.952
2010–2011	0.987	0.137	0.926	0.991	0.947	0.096	0.948	0.957
2011–2012	0.994	0.235	0.985	0.999	0.991	0.026	0.991	0.998
2012–2013	0.999	0.988	0.920	0.920	0.976	0.967	0.609	0.968
2013–2014	0.995	0.200	0.933	0.927	0.938	0.005	0.613	0.963
2014–2015	0.993	0.280	0.999	0.999	0.984	0.126	0.996	0.999
2015–2016	0.953	0.800	0.998	0.988	0.713	0.829	0.982	0.982
Mean	0.973	0.568	0.942	0.962	0.934	0.436	0.897	0.918

**Table 8:** Standard deviation of LAI_dfs_ for 10 years in each growth period.

Growing Stage	SD-LAI_dfs_ (×10^−4^)	
	A	B
Tillering	1.57	2.02
Booting	2.17	2.51
Flowering	3.21	4.2
Ripening	2.859	3.16

LAI_dfs_: The slope ratios of the line fitting equation of LAI_d._
